# *Tetrahymena* basal bodies

**DOI:** 10.1186/s13630-016-0022-8

**Published:** 2016-01-19

**Authors:** Brian A. Bayless, Domenico F. Galati, Chad G. Pearson

**Affiliations:** Department of Cell and Developmental Biology, University of Colorado–Anschutz Medical Campus, 2801 E. 17th Ave, Aurora, CO 80045-2537 USA

**Keywords:** *Tetrahymena*, Ciliate, Basal body, Centriole, Microtubule

## Abstract

*Tetrahymena thermophila* is a ciliate with hundreds of cilia primarily used for cellular motility. These cells propel themselves by generating hydrodynamic forces through coordinated ciliary beating. The coordination of cilia is ensured by the polarized organization of basal bodies (BBs), which exhibit remarkable structural and molecular conservation with BBs in other eukaryotes. During each cell cycle, massive BB assembly occurs and guarantees that future *Tetrahymena* cells gain a full complement of BBs and their associated cilia. BB duplication occurs next to existing BBs, and the predictable patterning of new BBs is facilitated by asymmetric BB accessory structures that are integrated with a membrane-associated cytoskeletal network. The large number of BBs combined with robust molecular genetics merits *Tetrahymena* as a unique model system to elucidate the fundamental events of BB assembly and organization.

## Introduction: the organism

**Fig. 1:**
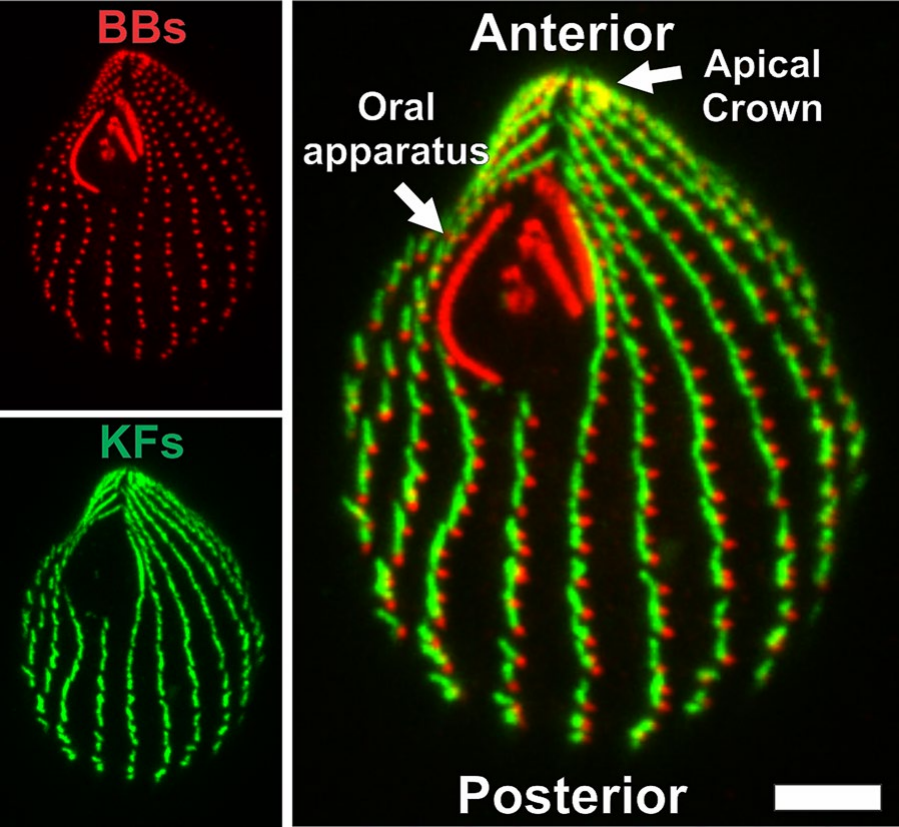
Polarized organization of *Tetrahymena* BBs. BBs are labeled in *red* (α-centrin, [27]) and kinetodesmal fibers are labeled in *green* (α-KF, [44]). The *merged image* highlights the organized ciliary array, the oral apparatus, and the apical crown which demarcates anterior–posterior polarity. *Scale bar* 5 μm

*Tetrahymena thermophila* is a free-swimming ciliate that utilizes hundreds of motile cilia for hydrodynamic force-generation. *Tetrahymena* belong to the superphylum Alveolata which also contains the parasitic Apicomplexans and the aquatic Dinoflagellates and together compose one of the largest groups of the kingdom Protozoa [1]. *Tetrahymena* are relatively large ovoid (20 μm wide and 35 μm long) single cells that contain 18–21 longitudinal rows of regularly spaced cilia (~30 per row; Fig. 1). Each cilium is nucleated and stabilized by a conventional basal body (BB). In addition, a single ciliated feeding structure, called an oral apparatus, contains 150 BBs segregated into four membranelles (tetra–“four’’ hymena–“membrane’’) and defines the organism’s anterior–posterior polarity. These cells divide every 3 h in a process that requires massive BB duplication to ensure that each daughter cell inherits an equal complement of cilia. *Tetrahymena* genetics allow for the generation of genomic knock-outs, knock-ins, and inducible promoter systems. Additionally, a sequenced and annotated genome was recently published [2]. With sophisticated molecular genetics, defined axes of organismal polarity and a tightly controlled linear arrangement of duplicating BBs, *Tetrahymena* is an outstanding cellular model for investigating the basic mechanisms of polarized BB assembly, stability, and organization.

## Basic *Tetrahymena* basal body structure

**Fig. 2:**
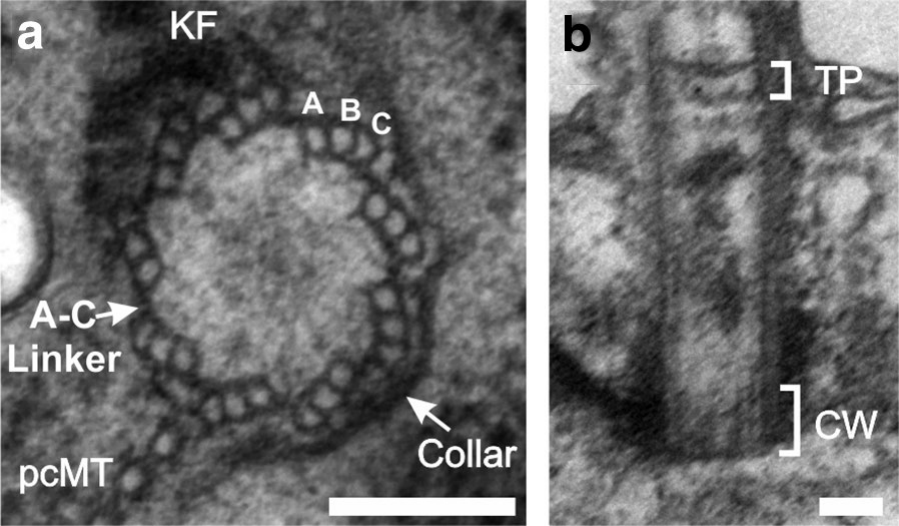
*Tetrahymena* BB structure. **a** Cross-sectional view of a proximal section of a *Tetrahymena* BB. *Collar* electron-dense collar; *pcMT* post-ciliary microtubules; *KF* kinetodesmal fiber; **b** longitudinal view of a BB; *TP* terminal plate; *CW* Cartwheel. *Scale bars* 100 nm

**Fig. 3:**
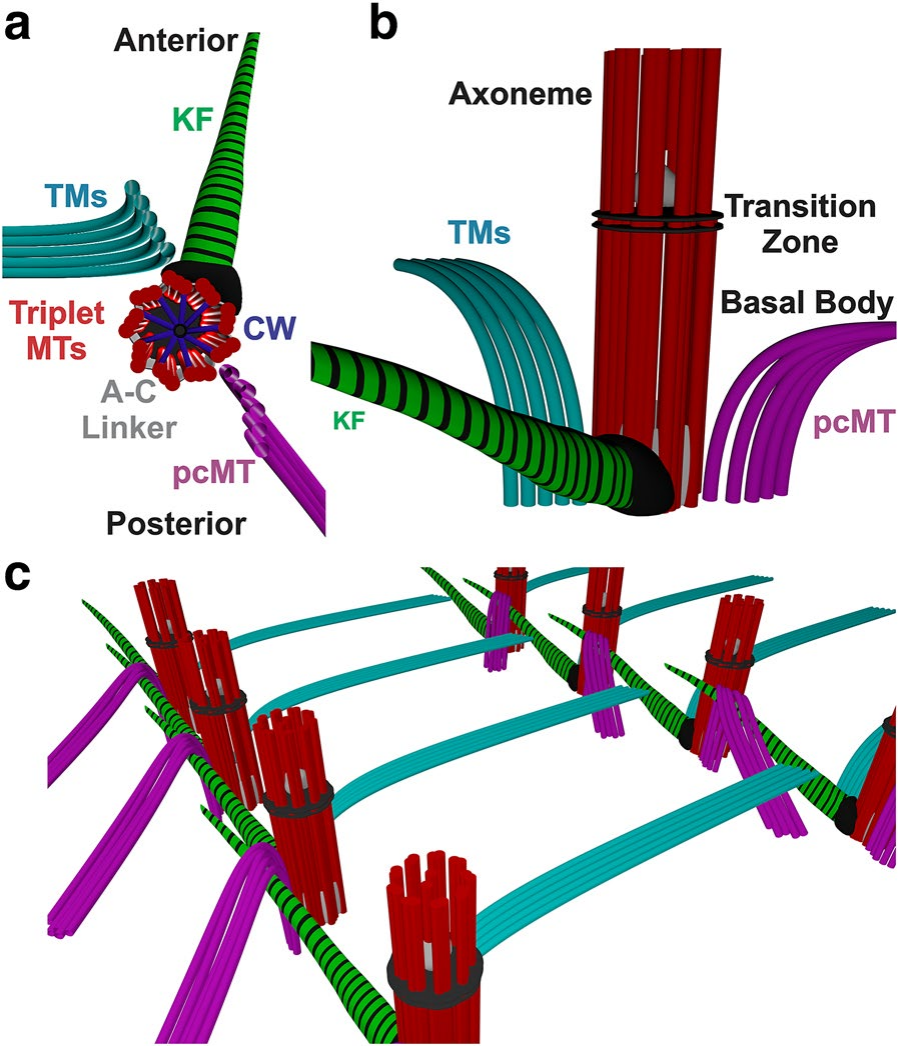
Schematic representation of *Tetrahymena* BBs and associated accessory structures. **a** 3D schematic of an individual cortical BB viewed from the interior of the cell. **b** An individual cortical BB viewed slightly offset from the anterior direction. **c** Image shows a portion of two ciliary rows highlighting the positioning of the three major accessory structures relative to neighboring BBs. *pcMTs* post-ciliary microtubules; *KF* kinetodesmal fiber; *TMs* transverse microtubules; CW cartwheel

*Tetrahymena* BBs are structurally similar to BBs in other eukaryotes. Mature *Tetrahymena* BBs are 500–600 nm in length and 180–220 nm in diameter [3]. The length of the BB comprises the typical triplet microtubule blades that are arranged into a cylinder with ninefold radial symmetry (Fig. 2a). The proximal end of the BB possesses three structures that establish and maintain the cylindrical organization. First, the A- and C-tubules of adjacent triplet microtubules are connected by an A–C linkage (Fig. 2a). Second, the proximal 60–90 nm of the BB contains a cartwheel structure composed of a central hub and nine spokes that connect to the A-tubule of each triplet microtubule blade (Fig. 2b). Importantly, the cartwheel is retained through the BB lifecycle, perhaps to ensure BB stability as these BBs must resist mechanical forces from beating cilia. Third, an electron-dense “collar” asymmetrically wraps around one side of the triplet microtubules (Fig. 2a). Above the cartwheel, the BB lumen encloses an electron-dense structure whose function remains poorly understood (Fig. 2b; [3]). The distal end of the BB is capped by the terminal plate (the *Tetrahymena* transition zone), which consists of two electron-dense opaque sheets that cross the lumen of the BB (Fig. 2b; [3]). While the core structure of the BB is largely conserved across phylogeny, ciliates, including *Tetrahymena*, utilize a unique assemblage of accessory structures that position and anchor BBs at the cell cortex.

## Additional BB structures or accessory structures

*Tetrahymena* BBs are endowed with accessory structures that coordinate BB positioning with cellular polarity and stabilize them against cilia-generated forces (Fig. 3). The location and composition of these structures depend on the BB population in the *Tetrahymena* cell. At the cell’s anterior pole, a ring of two closely positioned BBs, called dikinetids, begin each ciliary row and are associated with filaments of unknown composition called the apical filament ring [4]; together these structures are called the apical crown (Fig. 1). Within the oral apparatus, a dense microtubule meshwork organizes approximately 150 BBs into its four membranelles (Fig. 1; [5]). The majority of *Tetrahymena* BBs, however, are the cortical basal bodies that are required for cellular locomotion. Cortical BBs possess three major accessory structures: the post-ciliary microtubules, the transverse microtubules, and the kinetodesmal fiber (Fig. 3; [3]). Post-ciliary microtubules nucleate from the BB posterior face and radially project toward the posterior BB situated in the same ciliary row. Transverse microtubules originate from the BB anterior face and project upward and leftward (from the cell’s perspective) toward the cell cortex, where they overlap with the post-ciliary microtubules of anterior BB in the adjacent ciliary row. The kinetodesmal fiber is a striated structure that extends from the BB’s anterior face to the plasma membrane adjacent to the distal end of the anteriorly positioned BB within the same ciliary row. The kinetodesmal fiber also associates with the anterior BB’s post-ciliary microtubules [3]. By providing points of contact with the subcortical cytoskeletal network and neighboring BBs, accessory structures help establish and maintain the cellular organization and stability of BBs [3]. Moreover, these structures guide the placement of newly assembled BBs, suggesting that cortical BB accessory structures play an important role in cortical BB duplication [3, 6–8].

## Basal body origins

*Tetrahymena* cortical BBs arise next to existing BBs in what is called centriolar BB assembly. During assembly, a daughter BB forms orthogonally to a defined triplet microtubule at the anterior face of the proximal end of an existing mother BB [3]. New assembly commences with the formation of the cartwheel and a ring of short microtubules (called a pro-BB) that is separated from the mother BB by an amorphous electron-dense cloud [3]. As the pro-BB separates from the mother BB, the triplet microtubules elongate and tilt toward the apical surface to dock the BB distal end with *Tetrahymena*’s subcortical cytoskeletal network [3]. The pro-BB is positioned by the asymmetric localization of accessory structures on the mother BB, including the kinetodesmal fiber, which ensures that the new BB is appropriately spaced and positioned within the ciliary row [3]. Although cortical BBs assemble via the centriolar pathway, the origin of oral apparatus BBs is unclear and may arise from *de novo* assembly. Importantly, oral apparatus BB orientation, which is random early in development, coincides with BB linkage to an underlying microtubule network, representing a likely parallel to the process of BB orientation in vertebrate multi-ciliated cells [5, 9–14].

## Basal body life cycle and other functions

*Tetrahymena* undergo a closed mitosis where BBs do not function as centrioles in organizing a centrosome but rather remain docked at the cell cortex to organize cilia for the entire cell cycle. During mitosis, the two nuclei of *Tetrahymena* utilize distinct mechanisms to organize the microtubules of the mitotic micronucleus and amitotic macronucleus [15–19]. The micronuclear spindle microtubules are organized by a laminar structure analogous to the yeast spindle pole body while the macronuclear microtubules are nucleated from the nuclear envelope by a mysterious mechanism [20]. Importantly, because *Tetrahymena* BBs are solely used for locomotion and not mitosis, BB defects can be studied without perturbations that result in checkpoint arrest phenotypes. Existing mother BBs serve as sites of new BB assembly that occurs continuously throughout the cell cycle and increase in frequency before cell division [21–24]. The production of new BBs and their remarkably consistent integration into the polarized cell must be coupled with the dynamic and spatially controlled incorporation of proteins required for BB assembly.

## Basal body components

*Tetrahymena* BBs are molecularly conserved with the BBs and centrioles of other eukaryotes. Forward and reverse genetic approaches have been used in *Tetrahymena* to discover and elucidate the molecular mechanisms of important BB components [25–28]. Furthermore, purified BBs from *Tetrahymena* were used in combination with proteomics and immuno-electron microscopy to identify and localize many BB components to their ultrastructural BB domains [29]. These studies highlight *Tetrahymena* as a powerful model system to study the molecules and mechanisms of basal body assembly and function.

The triplet microtubules are composed of canonical α and β tubulin, while γ tubulin and ε tubulin are required for BB assembly and maintenance [30–32]. In addition, the *Tetrahymena* genome possesses δ tubulin along with the ciliate specific η and κ tubulins, although the functions of these isoforms remain unclear [2]. Also present are the conserved UNIMOD proteins (SAS-6, CEP135/Bld10, and SAS-4/CPAP) in addition to other conserved proteins like POC1 and members of the centrin family [27–29, 33]. Overall, the molecular conservation of BB components combined with adaptable genetics has led to a number of novel BB findings.

## Notable basal body findings

*Tetrahymena* has played a foundational role in our understanding of BB assembly, stability, and organization. Early studies capitalized on the polarized morphology of *Tetrahymena* BBs to study the propagation and maintenance of pre-existing BB order in the cell, which extended the pioneering studies of *Paramecium* ‘structural inheritance’ by Beisson and Sonneborn into other organisms [34, 35]. By mechanically inverting ciliary rows, Joseph Frankel and colleagues demonstrated that the *Tetrahymena* cortical architecture contains the epigenetic cues for placing new BBs within the polarized cell [35]. More recently, molecular–genetic and cytological studies identified a novel role for γ tubulin in regulating BB assembly [32]. Microtubule post-translational modifications are important for MT control and *Tetrahymena* was fundamental in the discovery and characterization of the MEC-17/α-TAT1 tubulin acetyl-transferase and the Tubulin Tyrosine Ligase-Like (TTLL) modifying enzymes that glutamylate and glycylate tubulin [36–40]. *Tetrahymena* has also played a large role in discovering a novel class of BB stability components and understanding their functions [27, 31, 41, 42]. Study of BB stability in *Tetrahymena* is advantageous because the cilia-generated forces experienced at the BB can be modulated experimentally [41]. *Tetrahymena’s* polarized cytology and ease of genetic manipulation have dramatically furthered our understanding of BB and tubulin biology.

## Conclusions: strengths and future of basal body research in *Tetrahymena*

Coupled with new high-resolution microscopy technologies, an expanding arsenal of molecular genetic tools make *Tetrahymena* an immensely powerful system for the next wave of BB research. The combined use of established forward genetics with Next-Generation sequencing enables the discovery of new molecules and mutants for further dissection of BB assembly and organization. BB protein localization and turnover dynamics are accessible to study in *Tetrahymena* using live cell imaging of fluorescently tagged proteins [29, 43]. Moreover, high-resolution light microscopy and cryo-electron tomography with the numerous and easily purified BBs of *Tetrahymena* will link the molecular and structural studies amenable to this system. The future is bright for BB research using this evolutionarily divergent model organism to understand the most highly conserved and divergent features of BB biology.

## Abbreviations

BB: basal body
